# Lauric Diacid‐Derived Sulfur‐Decorated Functional Polymers Displaying Programmable Thermal and Unconventional Luminescence Properties by Simple Thionation

**DOI:** 10.1002/marc.202500056

**Published:** 2025-07-31

**Authors:** Adam W. Woodhouse, Bercis Pektas, Cuong M. Q. Le, Jennifer A. Garden, Hatice Mutlu

**Affiliations:** ^1^ Institut de Science des Matériaux de Mulhouse UMR 7361 CNRS/Université de Haute Alsace Mulhouse Cedex France; ^2^ School of Chemistry Joseph Black Building Edinburgh UK

**Keywords:** aliphatic polythioesters, bio‐based, cluster‐triggered emission, polycondensation, post‐polymerization modification

## Abstract

Aliphatic polythioesters (featuring the (C═O)─S linkage) are recognized as useful complements to polyesters that possess intriguing properties, such as high optical features, metal coordination ability and affinity for metal surfaces, self‐healing capability, and improved crystallinity amongst others. Still, conventional synthetic approaches often require the use of toxic acyl chlorides. Thus, in this study, two semi‐crystalline polythioesters, **P1** and **P2**, were synthesized via a step‐growth polycondensation between a long‐chain bioderived diacid (1,12‐dodecanedioic acid, aka lauric diacid) and two commercially available dithiols, namely, 1,6‐hexanedithiol or 2,2’‐(ethylenedioxy)diethanethiol). The activation was achieved using 1,1’‐carbonyldiimidazole, which eliminates the need for acyl chlorides and leads to the formation of a useful by‐product, 1,8‐diazabicycloundec‐7‐ene imidazolium salt. An exemplary polythioester (i.e., **P2**) underwent a previously less‐reported post‐polymerization modification (hereafter referred to as modification, for clarity) with Lawesson's reagent to yield polydithioester **PP2**. This transformation induced a distinct change in material behavior, converting a semi‐crystalline structure (melting temperature: 67.7°C) into an amorphous one characterized by a glass transition temperature of −40°C, and significantly reducing its luminescent response. Thus, this study provides a more sustainable synthetic platform for the development of functional polythioesters with tunable thermal and optical properties.

## Graphical abstract

A versatile approach to synthesizing polythioesters using bioderived 1,12‐dodecanedioic acid and dithiol derivatives with 1,1'‐carbonyldiimidazole as the activating agent is reported. A key benefit is the generation of a valuable by‐product, 1,8‐diazabicycloundec‐7‐ene imidazolium salt, which has potential for reuse in other chemical applications. Additionally, post‐polymerization thionation reaction enables a straightforward tuning of the thermal and optical properties of the resulting polythioester.

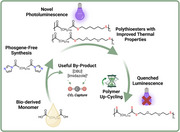

## 1 Introduction

As the demand for more sustainable polymers increases, most research efforts have focused on aliphatic polyesters, which can be bioderived and biodegradable [1]. Yet analogous polythioesters, where one of the oxygen atoms has been replaced by a sulfur atom, remain a relatively untapped source of potential [2]. Indeed, the incorporation of sulfur allows the polymer to exhibit a wide range of significantly differentiating characteristics, such as improved thermal properties [3], degradability [4], and higher refractive index [5] compared to their traditional ester counterparts. Therefore, polythioester synthesis represents a frontier in research, promising to pave the way for eco‐friendly alternatives to conventional polyesters. Crucially, the inclusion of a sulfur atom adjacent to the carbon‐oxygen bond makes polythioesters more susceptible to nucleophilic attack than polyesters, rendering them an attractive prospect as degradable materials [6]. Indeed, the intrinsically labile thioester bond can introduce dynamic covalent bond behavior by taking advantage of the reversible thiol‐thioester exchange reaction [7]. This behavior has been recognized as an effective strategy for developing covalent adaptable networks possessing intriguing properties, such as self‐healing [8], or of undergoing permanent and reversible state transitions from solid to fluid [9]. Thus, the inherent dynamicity of the thioester group can also be used to create polymers capable of reversible, orthogonal, and particularly, on‐demand degradation, offering the potential for circular polymer systems [10].

**FIGURE 1:**
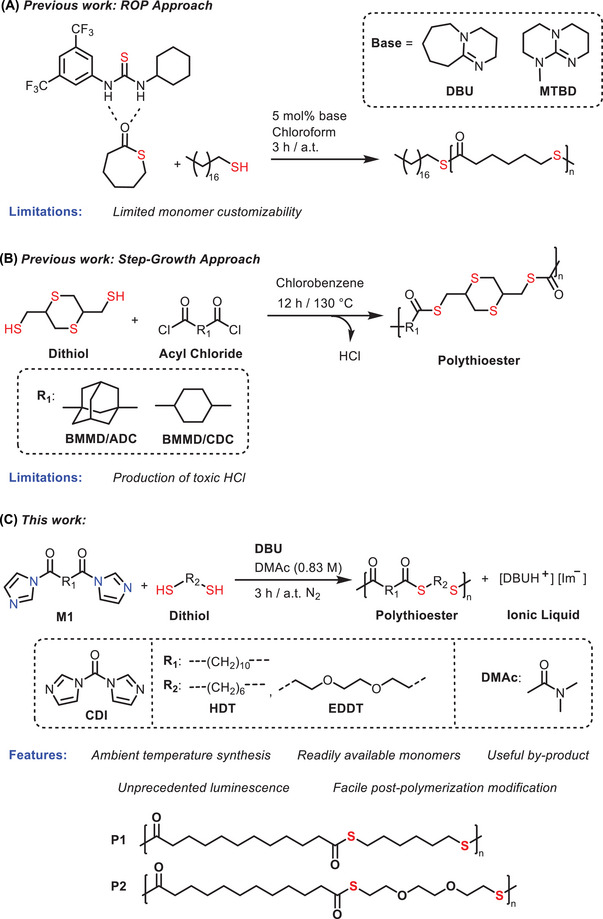
General scheme depicting the previously reported methods for synthesis of long‐chain aliphatic polythioesters (respectively A and B), in addition to the herein proposed methodology (C) which combines advantages of simple operation, no metals, mild conditions, and easily accessible monomers in order to deliver polymers **P1** and **P2**.

Aside from degradability, the improved thermal characteristics of polythioesters (compared to those of regular hydrocarbon‐based polymers) emphasize that they could represent a potential alternative to non‐renewable polyesters and open up a new class of circular materials [11, 12]. Indeed, aliphatic polythioesters are accessible via a variety of synthetic methods, such as the ring‐opening polymerization (ROP) of thiolactone, thionolactone, and dithiolactone derivatives, which usually results in reasonable molecular weight products under mild polymerization conditions (Figure 1A) [13]. While this strategy is facile and effective, the synthesis of cyclic monomers capable of ring‐opening polymerization currently requires tedious and cost‐ineffective conditions, and the respective polymerization is likely a consequence of the rapid intra‐ and interchain trans‐thioesterification resulting in an uncontrolled polymerization behavior. An alternate method to achieve aliphatic polythioesters involves the alternating ring‐opening copolymerization (ROCOP) of a variety of cyclic thioanhydrides and episulfides, which can be induced by simple organic ammonium salts [14]. However, these ROP and ROCOP methods are both limited to delivering polythioesters with a low number (3, 4, or 5) of carbon atoms in the repeating unit, as the synthesis of cyclic monomers with (extremely) long methylene spacers is frequently a formidable challenge [15].

In contrast, the step‐growth polycondensation of dithiols and acyl chlorides (Figure 1B) has the potential to prepare polymers with longer carbon chains in the repeat unit [16]. However, this methodology employs halogenated starting materials and releases HCl as a toxic by‐product. Unfortunately, research into step‐growth synthesis of polythioesters has mostly utilized such toxic reagents [15], overlooking the principles of Green and Sustainable Chemistry [17]. Thus, seeking an innovative and reliable methodology to construct sulfur‐decorated macromolecules from structurally diverse and commercially available starting materials has become a key goal in polymer science.

One emerging approach in polymer synthesis involves the use of reactive imidazole compounds as intermediates. In 2014, the stability and scalability of imidazole intermediates were demonstrated for small molecule synthesis, specifically for the formation of cyclic carbonates from commercially available and easy‐to‐handle 1,1’‐carbonyldiimidazole (CDI) [18]. More recently, bis‐carbonylimidazolide (BCI) monomers derived from CDI have been used to synthesize aliphatic, isocyanate‐free polyurethane derivatives via a solvent‐free and highly versatile approach [19, 20, 21], as well as sulfur‐containing aliphatic polydithiocarbonates that displayed non‐conventional intrinsic luminescence [22].

To align with the principles of more sustainable polymer design, the microbial fermentation‐derived 1,12‐dodecanedioic acid (also known as lauric diacid, LDA, Figure 1C) [23] was selected as the bio‐based precursor for the synthesis of BCI derivative. Long‐chain aliphatic monomers (≥C8) are receiving particular interest due to their capacity to enhance polymer crystallinity and thermal stability [24]. For example, the polyester‐6,12, synthesized from LDA and 1,6‐hexanediol, exhibits a higher melting temperature, *T*_m_, (88°C) compared to polyester‐6,4 (68°C) [25]. Indeed, a literature survey also reveals that such long‐chain aliphatic polymers decorated with functional units (e.g. esters, carbonates, etc.) could be considered as promising alternatives to polyethylene. Building upon this foundation, the polycondensation reaction of the dithiols (1,6‐hexanedithiol (HDT) or 2,2’‐(ethylenedioxy)diethanethiol (EDDT)) with the LDA‐derived BCI monomer was explored in the presence of the commercially available and widely used organic base 1,8‐diazabicycloundec‐7‐ene (DBU). Notably, DBU may also be isolated from the sea sponge, *Niphates digitalis*, thus highlighting its potential recognition as a renewable auxiliary. A key advancement demonstrated in this study is the post‐polymerization modification of the polythioester backbone through thionation using Lawesson's reagent. This transformation occurs without degradation of the polymer backbone and enables substantial alteration of the polymer properties. Thermal and optical characterization of the resulting polydithioester derivatives revealed significant changes in crystallinity and a reduction in intrinsic luminescence, thereby illustrating the potential for tailoring functional behavior through backbone editing. Overall, this work expands the synthetic toolbox for sulfur‐rich, sustainably derived polymers and highlights a modular route to property‐tunable polythioesters.

## 2 Results and Discussion

**FIGURE 2:**
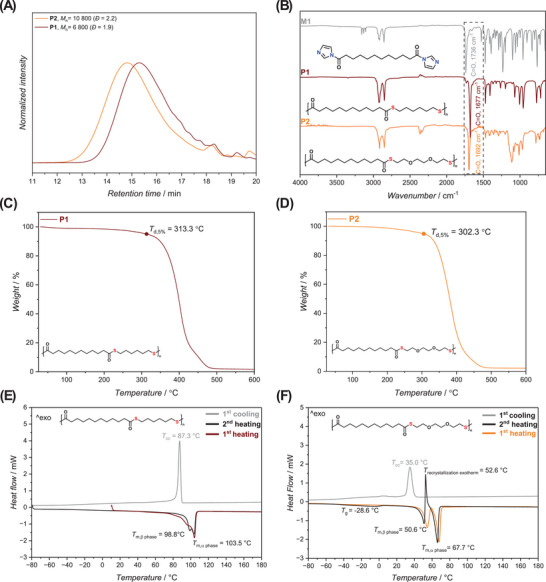
Physicochemical characterization of polythioesters **P1** and **P2**. (A) SEC elugrams (PS‐calibrated, THF, 35°C) showing apparent molar mass distributions: **P1** (*M*_n_ = 6800 g mol^−1^, *Đ* = 1.9) and **P2** (*M*_n_ = 10 800 g mol^−1^, *Đ* = 2.2). (B) FTIR spectra of monomer **M1**, and polymers **P1** and **P2**, highlighting the carbonyl shift from 1736 cm^−1^ (**M1**) to 1677 cm^−1^ (**P1**) and 1692 cm^−1^ (**P2**), indicative of thioester formation. (C,D) Thermogravimetric analysis (TGA) of **P1** and **P2**, showing excellent thermal stability with *T*_d,5%_ values of 313.3°C and 302.3°C, respectively. (E,F) Differential scanning calorimetry (DSC) curves of **P1** and **P2** displaying thermal transitions over two heating cycles. **P1** exhibits a melting point (*T*_m_) of 103.5°C and cold crystallization (*T*_cc_) at 87.3°C, while **P2** shows a glass transition (*T*_g_) at −28.6°C, cold crystallization at 35.0°C, and melting at 67.7°C.

Dithiols, while reactive, are not sufficiently nucleophilic to trigger thioester formation with a diacid [26]. Therefore, the diacid must first be activated with a suitable leaving group. Previous methods reported in the literature utilized acyl dichloride derivatives, bringing the disadvantage of producing hydrochloric acid as a toxic by‐product [27, 28]. 1,1’‐CDI was therefore selected as an activating agent to synthesize BCI monomers, for several reasons. Compared with short‐chain biomass‐derived aliphatic acids, LDA is more suitable for the preparation of BCI and the respective polythioester derivatives, as short‐chain aliphatic acids tend to stimulate intramolecular condensation [29]. Nevertheless, it should be noted that a number of carboxyl‐terminated monomers are also suitable, through use of the equivalent protocol. Accordingly, from initial screening experiments based on modified existing literature, it was found that the hitherto unreported BCI product of LDA (**M1**) was obtained in 90.3% isolated yield upon reacting 1.0 eq. of LDA together with 2.1 eq. of CDI in dimethyl acetamide (DMAc_dry_) for 2 h at ambient temperature under continuous inert gas flow. The successful synthesis of **M1** was confirmed primarily by Fourier‐transform infrared (FTIR) spectroscopy (Figures 2B and S1). Notably, the distinctive stretching vibration corresponding to the carbonyl C═O shifted to a higher wavenumber in **M1** (1736 cm^−1^) compared to pure LDA (1686 cm^−1^), which is attributed to the delocalization of the deprotonated hydroxyl anion of LDA reducing the double bond character of the carbonyl group. The inclusion of imidazole leaving groups in **M1** was also confirmed by the appearance of stretching bands at 1472 cm^−1^ (C─N), 1525 cm^−1^ (N═C─N) and ∼3150 cm^−1^ (the C─H stretching vibration of the carbon‐carbon double bond in the imidazole ring).

Furthermore, ^1^H NMR analysis of **M1** (Figure S2) displayed a triplet resonance at 2.82 ppm, corresponding to the CH_2_ adjacent to the carbonyl on the activated diacid. In general, compared to the LDA starting material, the magnetic resonances of the activated monomer shifted downfield due to the increased deshielding through the resonance effect of the imidazole ring. Crucially, the appearance of additional magnetic resonances in the δ = 2.0–3.0 ppm region indicates partial cleavage of the imidazole moieties, likely forming the corresponding diacid derivative. This degradation occurs under standard NMR conditions, emphasizing the sensitivity of the imidazole group to trace moisture and the need for careful sample handling. Still, by taking the ratio of the integrals of magnetic resonances attributed to **M1** and the unreacted diacid, residual DMAc and imidazole, the purity of the activated diacid was calculated to be 93.8%. Encouraged by the straightforward and scalable nature of the activated diacid synthesis, **M1** was subjected to polycondensation to synthesize polythioester derivatives. The thiol comonomers, 1,6‐HDT and 2,2’‐EDDT, were chosen for their structural features and proven performance in related sulfur‐containing polymers. HDT, a linear aliphatic dithiol, is industrially relevant – used in adhesive synthesis [30] and membrane fabrication [31], and even as a flavoring agent in meat‐based formulations [32]. Its regular structure is expected to promote crystallinity and flexibility. Conversely, EDDT introduces polar ether linkages into the polymer backbone, increasing chain mobility and solubility through disruption of ordered packing. Both dithiols are inexpensive, readily available, and reactive under mild conditions, making them ideal for exploring how dithiol structure affects the resulting polymer properties. Their previous application in polydithiocarbonate synthesis [22] enables direct comparison between sulfur‐containing polymers differing in backbone chemistry – thioester versus thiocarbonate. Accordingly, two structurally distinct aliphatic polythioesters **P1** and **P2** were targeted (as depicted in Figure 1C) under modified conditions adopted from a previously reported procedure for the synthesis of a small molecule thioester derivative. The synthetic design aimed to create tunable, potentially degradable sulfur‐containing polymers with varied polarity and flexibility. **P1** features extended methylene sequences and thioester linkages, while **P2** integrates ethylene glycol‐derived segments to impart polarity and enhance segmental mobility. This comparison underpins the modularity of the CDI‐mediated polymerization platform, which would allow precise adjustment of material properties by combining diverse diacid and dithiol building blocks. Subsequently, the polymer synthesis was accomplished by reacting **M1** with HDT or EDDT (1.0 eq.) and DBU (2.1 eq.) for 3 h in DMAc (compare Section S1.4), to yield polymers **P1** and **P2**, as white solids in good yields (up to 72.9%). The apparent number‐average molecular weights (*M*_n_) of 6800 g mol^−1^ (*Đ* = 1.9) and 10 800 g mol^−1^ (*Đ* = 2.2), respectively, were determined for **P1** and **P2** by polystyrene (PS)‐calibrated SEC in THF (Figure 2A and Table 1, Entry 1). However, it is important to note that both polymers exhibited limited solubility in common SEC solvents, including THF. Consequently, the reported molecular weights reflect only the soluble polymer fractions, and likely underestimate the true molecular size and dispersity of the bulk materials. Furthermore, PS‐calibrated SEC measurements are known to overestimate the actual molecular weight by approximately 50% for aliphatic polyesters such as poly(ε‐caprolactone) and poly(lactic acid) [33]. This should be taken into account when comparing absolute molecular weights of the synthesized polythioester derivatives.

**TABLE 1:** The number‐average molecular weight (*M*_n determined from the THF‐soluble fractions_), dispersity (*Đ*), melting point (*T*_m_), glass transition temperature (*T*_g_) and temperature of 5% degradation (*T*_d,5%_) values of polythioesters **P1** and **P2**, and the post‐polymerization modified polydithioester **PP2**. **PE1**, the polyester equivalent to **P1**, is also shown for comparative purposes [35].

Polymer	*M*_n_ (g mol^−1^)^a^	*Ð* ^a^	*T*_m_ (°C)^b^	*T*_g_ (°C)^b^	*T*_d,5%_ (°C)^c^
P1	6800	1.9	98.8 (α‐transition) 103.5 (β‐transition)	—	313.3
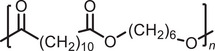 PE1	47 900	1.8	78.7	—	385.2
P2	10 800	2.2	54.3 (α‐transition) 67.7 (β‐transition)	−28.6	302.3
PP2	8750	2.4	—	−40.0	252.3

^a^ Determined by SEC measurements in THF with polystyrene standard, at a temperature of 35°C. *Đ* is defined as *M*_w_/*M*_n_.

^b^ Determined by DSC measurements from the second heating cycle at a scan rate of 10°C min^−1^, under a nitrogen atmosphere.

^c^ Determined by TGA measurements.

Throughout the reaction, the temperature was gradually increased from 50°C to 60°C to counteract the solidification of the mixture, which was presumably caused by the progressive increase in molecular weight and chain entanglement of the growing polymer. Crucially, the reactions were performed under a nitrogen atmosphere to prevent the formation of polydisulfide via oxidation of the deprotonated thiol monomers. DBU was selected as the base to deprotonate the thiol, as it was previously reported to be an efficient promoter for the synthesis of diverse thiol‐derived polymers via alternative condensation polymerization routes. DBU is removed from the reaction mixture by scavenging the formed by‐product imidazole by yielding an ionic liquid, i.e. DBU/imidazole (vide infra) [22]. This side product formation serves not only as a practical purification step but also exemplifies the valorization of the side product, aligning with the Green Chemistry Principles of waste prevention *(Principle 1*) and design for degradation and reuse (*Principle 10*). Moreover, the resulting ionic liquid is known to reversibly absorb carbon dioxide, offering an additional sustainability advantage by integrating CO_2_ capture capability into the reaction design [34], further enhancing the sustainability profile of the process by contributing to *Principle 7*: use of renewable feedstocks and *Principle 11*: real‐time analysis for pollution prevention, if used for integrated capture and conversion. The multifunctionality of the DBU/imidazole ionic liquid thus transforms a potential waste stream into a value‐added, CO_2_‐absorbing co‐product, demonstrating a holistic, green‐by‐design approach to polymer synthesis. It is important to highlight that the reported synthesis of the oxygen‐containing polyester analogue of **P1** (**PE1**, Table 1) involves significantly harsher conditions [35]. The process follows a two‐step melt polycondensation, where 1,12‐dodecanedioic acid and diols are first esterified at 180°C–190°C for 2–3 h under nitrogen, followed by polycondensation under high vacuum (50 Pa) at 210°C–240°C for 4–5 h using Sn(Oct)_2_ (1.0 wt.%) as a catalyst. These high‐temperature, low‐pressure conditions not only demand specialized equipment but also pose challenges for scalability and energy efficiency. In stark contrast, the synthesis of polythioesters **P1** and **P2** proceeds under much milder conditions, i.e., below 65°C, in solution, and without the need for vacuum or continuous by‐product removal, demonstrating a more energy‐efficient and operationally accessible approach, aligned with the principles of Green Chemistry and process intensification.

To verify the successful polymerization of monomer **M1** into polythioesters **P1** and **P2**, FTIR and NMR spectroscopy were employed. Comparison of the FTIR spectra of **M1** and **P1** showed a shift of the carbonyl carbon band from 1736 to 1677 cm^−1^, indicating a change in the electronic environment of the carbonyl group upon formation of the thioester bond (Figure 2B). A similar trend was observed in the FTIR spectrum of **P2**. Further structural validation was provided by NMR analysis (Figure S4). For **P1**, a key triplet resonance at 2.83 ppm was assigned to the methylene protons adjacent to the thioester carbonyl. This resonance is downfield‐shifted compared to the corresponding protons in the diacid precursor (LDA), which appear at 2.28 ppm. The increased chemical shift is attributed to the reduced resonance stabilization in the thioester group, which renders the carbonyl carbon more electron‐deficient and thus more deshielding. In contrast, the carboxylic acid group in the precursor benefits from enhanced resonance delocalization, resulting in greater shielding and a lower chemical shift. Similar trends were observed for **P2** (Figure S4). The ^13^C NMR spectra of **P1** and **P2** (shown in Figures S5 and S6) have also confirmed the successful polythioester formation. For **P1**, the thioester carbonyl resonance appears at 199.8 ppm, while for **P2**, it is slightly downfield at 199.2 ppm. The conversion to polymer was calculated to be 92.9% and 93.9% for **P1** and **P2**, respectively, based on the integration of diagnostic magnetic resonances corresponding to **M1** and the polythioester. Interestingly, over the course of the reaction, a white residue formed on the N_2_ outgassing needle. ^1^H NMR analysis of this residue displayed magnetic resonances consistent with those observed for a DBU/imidazole ionic liquid, confirming the generation of this compound as a by‐product (Figure S7).

The thermal properties of the polymers were investigated by thermogravimetric analysis (TGA) and differential scanning calorimetry (DSC). Importantly, TGA and derivative thermogravimetric (DTG) profiles, as shown in Figure 2C,D and Figures S8 and S9, clearly support the formation of high‐molecular‐weight materials, as evidenced by the sharp, well‐defined degradation transitions and the high thermal decomposition temperatures (*T*_d,5%_) with values of **P1** and **P2** shown to be 313.3°C and 302.3°C, respectively (Table 1). Here, the increased susceptibility of **P2** toward degradation is attributed to the increased flexibility of the carbon‐oxygen bonds from the EDDT linkages. It is crucial to emphasize that **P1** and **P2** both displayed *T*_d,max_ (a maximum rate decomposition temperature) higher than 350°C. As a result, these materials can be processed safely without thermal degradation at temperatures 30°C–40°C higher than their corresponding *T*_d,5%_.

DSC analysis showed **P1** and **P2** to be semi‐crystalline (Figure 2E,F). Unlike the TGA analysis, which showed that the *T*_d,5%_ values of the two polythioesters are similar, DSC analysis reflected that the melting points of **P1** and **P2** differ by up to 44°C. For the HDT‐containing polymer **P1**, two sharp endothermic melting points were observed: an α‐transition at 98.8°C and a β‐transition at 103.5°C. These two melting points correspond to the domains relating to the diacid‐derivative and dithiol monomers. The β‐transition likely corresponds to the diacid domain, as it has stronger Van der Waal's intermolecular forces than the dithiol domain and a higher degree of crystallinity. The α‐transition, therefore, corresponds to the dithiol domain which has a shorter carbon chain. Interestingly, the *T*_m_ of **P1** is higher than the equivalent polyester (**PE1**, entry 2 in Table 1) synthesized from 1,6‐hexanediol and LDA, which displayed a *T*_m_ at 78.7°C [35]. This higher melting point shows the improved thermal properties of **P1** compared to the equivalent polyester. In polymer **P2**, similar α‐ and β‐transitions were observed at 54.3°C and 67.7°C, respectively. The melting points of **P2** were considerably lower than those observed in **P1** which can be explained by comparing the structures of the polymers. Compared to **P1**, **P2** contains two additional oxygen atoms in the backbone. It is known that ether links facilitate the rotation of neighboring methylene groups, resulting in an increase in flexibility, which could cause a lowering of the melting temperature of **P2**. Therefore, by simply altering the structure of the dithiol monomer, the melting points of the polymers can be effectively tuned. The polymers **P1** and **P2** also revealed strong and sharp peaks in the cooling scan, the cold crystallization temperature (*T*_c__c_) of 87.3°C and 35.0°C, respectively, indicating that the long‐chain polyesters had a distinct crystallization behavior and they were semi‐crystalline materials similar to polyethylene. Indeed, by comparing with other linear aliphatic polythioesters derived from 1,6‐diol and dicarboxylic acids with different carbon numbers [36], **P1** has higher *T*_m_ than those with shorter chain length in the dicarboxylic acids.

Interested in evaluating the mechanical properties of the polymers, the formation of polymers films by compression molding was investigated. Unfortunately, the resulting samples were substantially brittle and challenging to characterize due to the crystalline structure, highlighting the potential trade‐off between thermal stability and flexibility.

The potential for post‐polymerization modification of the polythioesters was investigated to observe the effect of further structural tailoring on their thermal properties. Furthermore, the facile chemical modification of polymers (i.e. polymer metamorphosis) is a desirable goal, as it creates the potential for the introduction of new phases of life by changing the polymer properties (upcycling) or improving depolymerizability [37, 38]. In other words, demonstrating the potential of post‐polymerization transformation is postulated to effectively extend the useful lifetime of recyclable polymers with the advantage of avoiding excessive chemical recycling, thereby reducing additional energy and material consumption.

**FIGURE 3:**
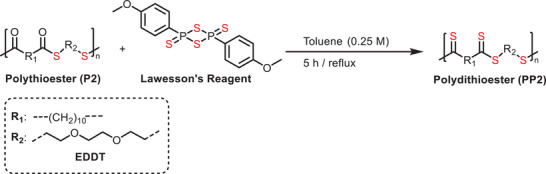
Reaction scheme demonstrating the post‐polymerization modification of polythioester **P2** to polydithioester **PP2**, as reported for the first time in this publication.

**FIGURE 4:**
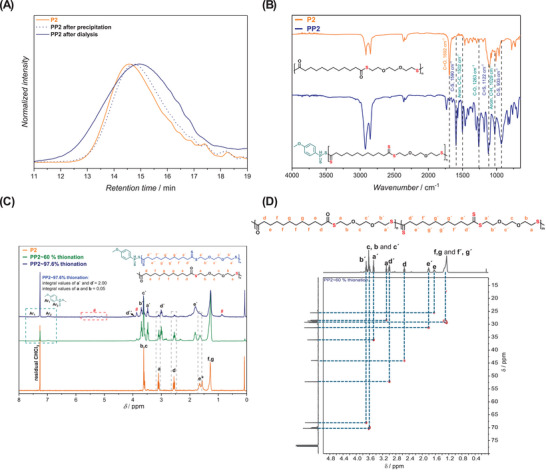
Comprehensive characterization of polythioester **P2** and its thionated derivatives **PP2** (∼60% and ∼97.6% thionation). (A) SEC traces of **P2**, **PP2** (∼97.6%) after precipitation, and **PP2** (∼97.6%) after dialysis, showing similar retention profiles but highlighting the importance of dialysis for removing low‐molecular‐weight species and improving purity. (B) FTIR spectra showing the disappearance of the carbonyl C═O stretch at 1692 cm^−1^ and the emergence of new bands in the 1025–1225 cm^−1^ range, indicative of C═S formation in **PP2** (∼97.6%). (C) Overlaid ^1^H NMR spectra (300 MHz, CDCl_3_) of **P2** (orange), **PP2–60%** (green), and **PP2–97.6%** (blue). The progressive downfield shift of methylene proton signals (e.g., a → a′, b → b′, d → d′) confirms increasing degrees of thionation. Aromatic signals marked with (#) indicate residual Lawesson's reagent by‐products, which diminish upon purification via dialysis. (D) 2D‐HSQC spectrum (500 MHz, CDCl_3_) of **PP2** (∼60% thionation) after purification via dialysis. The spectrum reveals distinct proton–carbon cross‐peaks corresponding to both thioester and dithioester segments, confirming their coexistence within the polymer backbone. Notably, no signals corresponding to terminal thiol or carboxylic acid groups are observed, indicating that the polymer chains remained intact and no degradation occurred during the thionation process.

The thionation of thioesters to dithioesters utilizing Lawesson's reagent is a well‐reported reaction in literature, yet so far this has been almost exclusively limited to small molecule transformations [39]. Notably, thioesters are also among the most challenging functional groups to thionate, due to their lower electrophilicity and the electron‐donating character of sulfur, which diminishes the reactivity of the carbonyl carbon toward thionation. As such, successful conversion of polythioesters into their polydithioester analogues represents a synthetically demanding and mechanistically significant advance. Recently, a study investigated the thionation of poly(2‐ethyl‐2‐oxazoline) using Lawesson's reagent, with an increased degree of thionation giving increased *T*_g_ values [40]. Still, the thionation of polythioesters remains unreported. In this study, **P2** was selected for modification over **P1** due to its superior solubility in toluene, which facilitates efficient reaction with Lawesson's reagent. To this end, the EDDT‐containing polythioester **P2** was refluxed with Lawesson's reagent in toluene (0.25 M) for 5 h, using an adapted literature procedure (Figure 3) [41]. Upon reaction with Lawesson's reagent, an immediate color change from white to orange was observed. Precipitation from ice‐cold MeOH followed by dialysis resulted in the isolation of a sticky orange polymer with a yield of 70.6%, with *M*_n, determined from the THF‐soluble fractions_ = 8750 g mol^−1^ and *Đ =* 2.4 (Table 1, Entry 4). The modification was successful, occurring with a minimal decrease (∼1300 g mol^−1^) in the weight‐average molecular weight (Figure 4A). Crucially, purification via dialysis was necessary to remove residual Lawesson's reagent and any potential small‐molecule side products, yielding a cleaner polymer sample suitable for further analysis. This step ensured that the observed polymer properties, such as thermal behavior, reflected the intrinsic nature of the polydithioester backbone, rather than artifacts from unreacted or degraded thionating agents.

The conversion of polythioester **P2** to polydithioester (**PP2**) was primarily confirmed via FTIR. In this case, the characteristic carbonyl C═O stretching band (1692 cm^−1^) has mostly disappeared due to conversion to C═S (Figure 4B). Furthermore, the fingerprint regions of the two spectra widely differed, with **PP2** showing a variety of bands in the fingerprint region (500–1600 cm^−1^). While the bands in this region are difficult to accurately assign, a sharp band with a stretching frequency of 1122 cm^−1^ was observed in the spectrum of **PP2**. This band could correspond to the C═S bond in the polydithioester, which is known to be in the range of 1025–1225 cm^−1^ [42].

^1^H NMR spectroscopy was used as a secondary tool to confirm the synthesis of polydithioester and to highlight the critical role of dialysis in removing residual reagents and by‐products, ensuring accurate and reliable characterization (Figure 4C and Figure S10). Compared to the polythioester, the magnetic resonances of the thionated product experienced a downfield shift. For example, the CH_2_ environment adjacent to the carbonyl carbon resulted in a magnetic resonance at 3.07 ppm in **P2**, which shifted to 3.48 ppm in **PP2**. The downfield shift is attributed to the improved resonance stabilization in **PP2** which reduces the electron density on the neighboring hydrogen environments. By taking the ratio of the magnetic resonances corresponding to polythioester and polydithioester, the conversion rate was calculated to be 97.6% (see the specified section within the Supporting Information), demonstrating the efficiency of the modification within a reasonable reaction time. To gain further insight into the post‐polymerization modification, we also intentionally targeted partial thionation (∼60%) of the polythioester **P2**, allowing the stepwise transformation to be monitored by ^1^H NMR spectroscopy. This approach enabled us to distinguish between the residual thioester and newly formed dithioester signals, thereby quantifying the extent of conversion. Particularly the 2D‐HSQC NMR analysis of **PP2** (∼60% thionation) obtained upon dialysis, shown in Figure 4D, provided detailed structural insight into the coexistence of thioester and dithioester functionalities within the polymer backbone. The spectrum displayed clear cross‐peaks correlating proton and carbon environments of both unmodified (thioester) and thionated (dithioester) segments. Specifically, the duplicate or triplet signal patterns for several proton environments, such as a/a`, b/b`, and c/c`, indicate that these methylene groups exist in chemically distinct environments depending on whether they are adjacent to a C═O or a C═S group. The shift of certain cross‐peaks to lower proton field (i.e., downfield) and lower carbon field for the dithioester units was consistent with increased deshielding effects due to enhanced resonance delocalization in the C═S functionality. Importantly, no additional cross‐peaks corresponding to terminal thiol (─SH) or carboxylic acid (─COOH) end groups were observed. This absence suggests that no significant backbone cleavage or degradation occurred during the thionation process, preserving the polymer chain integrity throughout the reaction. The clean correlation pattern confirms that the modification proceeds via selective thionation rather than undesired side reactions or chain scission.

The thermal properties of **PP2** (∼97.6%) were also analyzed via TGA, DTG and DSC analysis (Figures S11–S13), with the polymer displaying a significantly lower thermal stability than **P2** (*T*_d,5%_ = 252.3°C vs. 302.3, respectively) and displaying amorphous properties with a glass transition temperature of −40°C (i.e. the absence of melting points). This change from semi‐crystalline to amorphous behavior upon the post‐polymerization modification is likely due to weaker dipole‐dipole interactions between the polymer chains in the polydithioester compared to the polythioester, as the C═S bond has a lower dipole moment than C═O.

While our initial objective was to investigate the thermal properties of the synthesized poly(di)thioesters (given their potential as high‐performance, bio‐based materials) an unexpected UV absorption signal during SEC measurements of **P1** and **P2** prompted us to examine their photophysical behavior. This unforeseen observation motivated a preliminary investigation into their luminescence characteristics, which we present here as a novel and, to the best of our knowledge, unprecedented feature of polythioesters. Although exploratory in nature, our findings point to an exciting avenue for functional applications, particularly in the context of cluster‐triggered emission (CTE), and lay the groundwork for more comprehensive studies that are currently underway.

**FIGURE 5:**
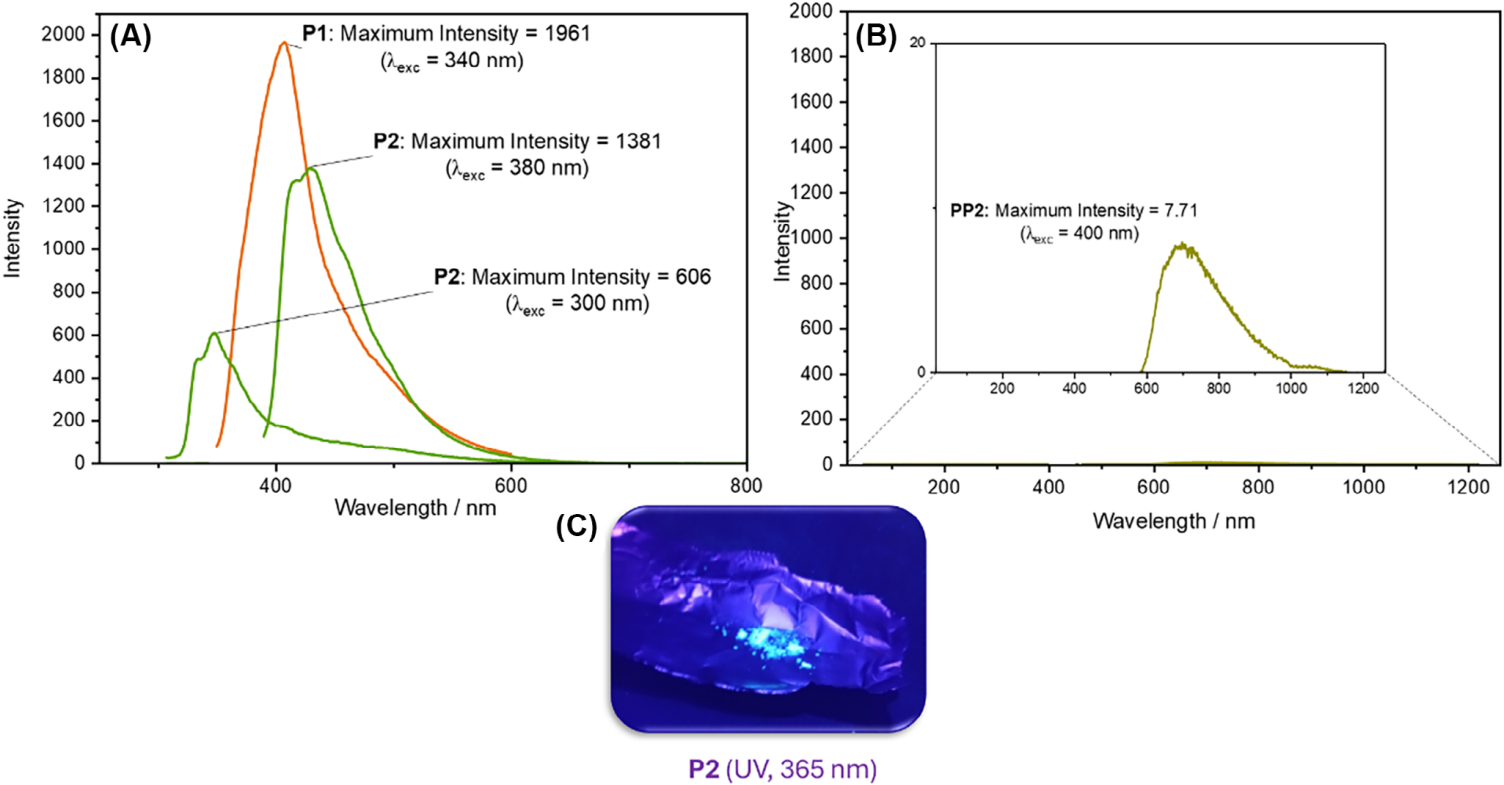
Fluorescence spectra (DCM, 40 mg·mL^−1^) of (A): polythioester **P1** (orange color), **P2** (green color), (B): polydithioester **PP2** (∼97.6% thionation), with the relative intensities labelled, and (C): photo of luminescence displayed by solid sample of **P2** under hand‐held UV light (365 nm) lamp.

Specifically, polymers **P1** and **P2** displayed striking luminescence upon irradiation with 365 nm UV light, both in dichloromethane (DCM) solution and the solid state, respectively (Figure 5). Such luminescence behavior has been reported in the literature for compounds lacking conventional chromophores, such as proteins, peptides, and rice [43], as well as recently for the sulfur‐containing polydithiocarbonates [22]. The phenomenon is hypothesized to be due to the clustering of electron‐rich functional groups and heteroatoms (e.g. O, N, S), resulting in effective through‐space conjugation and “clustered chromophores” with enriched energy levels and lowered energy gaps [44]. Notably, CTE can be enhanced by increasing polymer concentration. In some cases, greater rigidity may promote clustering of electron‐rich functional groups, although this is not a universal rule.

To elucidate the absorbance behavior of the polymers, UV–vis spectra were measured in DCM at 40 mg mL^−1^ (Figure S14). **P1** and **P2** both displayed one absorption peak at 230 nm, corresponding to a *π*–π* transition of the C═O bond [45]. In **PP2** (∼97.6%), a strong peak was observed at 306.5 nm, corresponding to the *π*–π* of the C═S bond, as well as a weak peak at 463 nm, assigned to the n‐π* transition [46]. It was noted that polymers **P2** and **P1** were both visibly luminescent when irradiated under a 365 nm UV handlamp.

Fluorescence spectrometry showed that for **P1**, a maximum emission was observed at 408 nm with an excitation wavelength (λ_exc_) of 340 nm (Figure 5A) [47, 48]. **P2** exhibited slightly weaker emission than **P1**, with peaks being observed at 417 and 428 nm with λ_exc_ = 380 nm, and a smaller peak observed at 348 nm for λ_exc_ = 300 nm. The maximum relative emission intensity of **P1** was 1961 arbitrary units (a.u.), while for **P2** the maximum intensity was 1381 a.u., showing that **P1** is more strongly luminescent than **P2**. This variance in luminescence intensity may arise from differences in polymer chain conformation and segmental flexibility, which can influence the proximity of heteroatoms even in dilute solution. Similarly, **PP2** displayed vastly reduced luminescence (7.7 a.u.), thus demonstrating the tunability of emission via post‐polymerization modification. The mentioned phenomenon is in line with reported examples in the literature, which postulate that thionation results in a substantial quenching of fluorescence by facilitating intersystem crossing to longer‐lived triplet states, thereby promoting phosphorescence. In addition, the bathochromic (red) shift observed in **PP2** can be attributed to the increased sulfur content introduced during thionation, which modifies the electronic structure of the polymer and reduces the HOMO–LUMO gap. Similar effects have been reported for other thionated polymers, including the work by Luxenhofer and co‐workers [40]. This feature opens up opportunities for repurposing/recycling of the polythioester depending on the desired function. To provide a quantitative measure of the photoluminescence properties, relative fluorescence quantum yields (*Φ*_F_) were determined using pyrene in dichloromethane (DCM) as the reference standard (*Φ*_F_ = 6.8%) (Figure S15) [49]. Under 340 nm UV irradiation, the emission of the **P1**/DCM solution was the brightest with a *Φ* value of 0.823 (refer to Section S1.2.5 for calculation details), while that of **P2**/DCM solution was much weaker with a *Φ* value of 0.082. These values quantitatively support the earlier qualitative observations that **P1** exhibited strong luminescence, particularly under 340 nm excitation. In contrast, **P2** showed excitation‐wavelength dependent emission, with a surprisingly high quantum yield at 380 nm (*Φ* = 2.51), suggesting potential for tunable photophysical behavior. The significantly reduced *Φ* of **PP2** (0.09) at 380 nm after thionation confirmed that the introduction of C═S bonds lead to substantial quenching of fluorescence, likely via enhanced intersystem crossing. These data confirmed the chemical responsiveness of the optical properties and underpinned the potential of this platform for luminescence control through structural editing.

## 3 Conclusions

We have developed a relatively green approach to prepare two polythioesters (**P1** and **P2**) from commercially available dithiols and a bioderived diacid. The process benefits from avoiding toxic halogenated reagents and yields a potentially repurposable by‐product in the form of a DBU/imidazole ionic liquid. Furthermore, the resulting polythioesters exhibit structure–property tunability (compared to their oxygen counterparts), with thermal behavior modulated by the nature of the dithiol (rigid vs. flexible), and optical behavior responsive to post‐polymerization chemical modification. We have also demonstrated the proof‐of‐concept for a chemical metamorphosis that a polythioester (**P2**) can be efficiently transformed into polydithioesters (**PP2**) through a simple thionation, leading to a shift from semi‐crystalline to amorphous morphology and a dramatic quenching of intrinsic luminescence. These transformations highlight the potential for stimuli‐responsive materials, where properties such as thermal behavior and photoluminescence can be tailored on demand. Indeed, the discovery of intrinsic photoluminescence was originally observed as an unexpected UV signal, which further expands the functional potential of these materials and is currently being explored in greater detail. Furthermore, the observed changes support the vision of polymer upcycling, where materials can undergo lifecycle extension or repurposing through mild, selective chemical editing, an approach relevant to future circular material strategies. While preliminary in scope, this study was designed as a concept‐driven extension of earlier work and lays the foundation for ongoing investigations into broader monomer scopes (i.e., diacids and dithiol derivatives) and chain‐end control. Although this study presents only two polythioesters, the contrasting structure–property relationships established by HDT and EDDT validate the robustness and tunability of the CDI‐mediated polycondensation strategy. Ongoing efforts focus on further exploring and comparing key characteristics of polythioesters, particularly the intrinsic reactivity of the weak (C═O)─S bond, with those of their polyester analogs, aiming to harness their greater potential for the development of dynamic and responsive materials.

## Conflicts of Interest

The authors declare no conflicts of interest.

## Supporting information




**Supporting file 1**: marc202500056‐sup‐0001‐SuppMat.pdf

## Data Availability

The data that support the findings of this study are available from the corresponding author upon reasonable request.
