# Supplementary material for: Lauric Diacid‐Derived Sulfur‐Decorated Functional Polymers Displaying Programmable Thermal and Unconventional Luminescence Properties by Simple Thionation
Source: Macromol Rapid Commun. 2025 Jul 31;46(23):e00056. doi: 10.1002/marc.202500056 (PMC12687712; doi:10.1002/marc.202500056)
Supplement: Supplementary file 1 — Supporting file 1: marc202500056‐sup‐0001‐SuppMat.pdf [file MARC-46-e00056-s001.pdf]

Supporting Information

Lauric diacid-derived sulfur-decorated functional polymers displaying programmable thermal and unconventional luminescence properties by simple thionation

A. W. Woodhouse, B. Pektas, Dr. Cuong M.Q. Le, Dr. J. A. Garden, Prof. Dr.rer.nat. H. Mutlu*

A. W. Woodhouse, Cuong M.Q. Le, B. Pektas, Prof. Dr.rer.nat. H. Mutlu

Institut de Science des Matériaux de Mulhouse, UMR 7361 CNRS/Université de Haute Alsace, 15 Rue Jean Starcky, Mulhouse Cedex 68057, France

E-mail: hatice.mutlu@uha.fr

A.W. Woodhouse, Dr. J. A. Garden

School of Chemistry, Joseph Black Building, David Brewster Road, Edinburgh EH9 3FJ, United Kingdom

1. **Experimental Section**

1.1 Materials

1,1’-Carbonyldiimidazole (CDI, Tokyo Chemical Industry), 1,8-diazabicyclo(5.4.0)undec-7-ene (DBU, Tokyo Chemical Industry), 1,12-dodecanedioic acid (LDA, Sigma-Aldrich), 1,6-hexanedithiol (HDT, Sigma-Aldrich), 2,2’-(ethylenedioxy)diethanethiol) (EDDT, Sigma-Aldrich) and Lawesson’s reagent (Sigma-Aldrich) were used as received. To ensure accurate stoichiometric balance, the masses of the reagents were adjusted to account for their purity. The masses shown below are therefore the calculated values before reagent purity was considered.

1.2 Characterization

1.2.1 Nuclear Magnetic Resonance (NMR) Spectroscopy

The ^{1}H NMR spectra were recorded on Bruker Avance (300 MHz or 500 MHz) spectrometer, while the 13C NMR were recorded on 75 MHz and 125 MHz. Spectra were referenced on residual solvent signal of $CDCl_3$ according to Nudelman et al: 7.26, and 77.16 ppm for ^{1}H and ^{13}C, respectively. Deuterated solvents were purchased from Euriso-TOP and used without further purification.

1.2.2 Fourier-Transform Infrared (FTIR) Spectroscopy

All IR measurements were performed on a Bruker Alpha ATR-IR Spectrometer with a range of 650 to 4000 cm^{-1} at ambient temperature.

1.2.3 Size Exclusion Chromatography (SEC)

The apparent number average molar mass (M_n) and the molar mass distribution [$Đ$ (dispersity index) = M_w/M_n] values of the polymers were determined using size exclusion chromatography (SEC) measurements on an Agilent 1260 Infinity instrument. The instrument is comprised of an autosampler, a set of columns composed of a guard column (50 × 7.5 mm) and two analytical columns (Polymer Laboratories ResiPore, 300 × 7.5 mm nominal particle size: 3 µm; porosity: 2 µm), G1314B variable wavelength detector operating at 280 nm, a G7800A multidetector suite consisting of a refractive index and a viscosimeter detector. The measurements were conducted with a flow rate of 1 mL/min at 35 °C using tetrahydrofuran as the eluent. The calibration was carried out by employing different linear poly(styrene) standards (EasiVial polystyrene standards from Agilent) ranging from 162 to 3.64×10^{5} g mol^{-1}. The polymer samples were dissolved in aforementioned eluent and filtered over a 0.2 μm filter prior to the measurement. Agilent GPC/SEC software and multi-detector were used to obtain the molecular weight data.

1.2.4 Ultraviolet-visible (UV-Vis) Spectroscopy

UV-Vis absorbance were recorded on a V730 UV-Vis spectrometer (Jasco Corporation, Japan) operating at 1.0 nm bandwidth, scan speed 1000 nm/min. Polymer was dissolved in dichloromethane and transferred to a quartz cuvette (4 × 1 × 1 cm) and spectra were obtained from 235 to 800 nm.

1.2.5 Fluorescence Spectroscopy

Fluorescence spectra were recorded on a FP-8200 (Jasco Corporation, Japan) spectrofluorometer equipped with a xenon lamp. The excitation and emission bandwidths (5 nm) and scan rate (1000 nm min^{-1}) were kept constant. The polymer was dissolved in suitable solvent and transferred to a quartz cell (4 × 1 × 1 cm) and the spectra were recorded without degassing.

The fluorescence quantum yield was calculated using pyrene in dichloromethane solution (Φ_F = 0.068) as a standard.[1,2] For consistency, absorption measurements for all samples were conducted under dilute conditions (abs ≤ 0.1) to prevent aggregation effects and ensure reliable quantum yield determinations. After that, the emission spectra for each sample and the pyrene reference standard were recorded. The area under the emission curve was integrated to calculate I. The integrated emission intensity represents the total fluorescence output across the entire emission wavelength range. Because dichloromethane was used as a solvent to dissolve both the polymer and reference standard; the refractive index correction was thus omitted in the calculation. The emission quantum yields for all polymers were calculated based on the following equation:

$$\Phi_{F,p} = \Phi_{F,\ ref} \times \frac{A_{ref}}{A_p} \times \frac{I_p}{I_{ref}} \quad (eqation\ 1)$$

Where:

- *ref* denotes the reference standard.
- *p* denotes the polymer sample.
- Φ_F is the fluorescence quantum yield.
- A is the absorption.
- I is the integrated emission intensity.

1.2.6 Thermogravimetric Analysis (TGA)

TGA data were recorded on a Thermogravimetrics METTLER - TOLEDO TGA / DSC 3+ from ambient temperature to 600 °C at 30 °C min^{-1} under nitrogen atmosphere.

1.2.7 Differential Scanning Calorimetry (DSC)

A Mettler Toledo DSC1 was used for thermal analysis. The DSC program was set from -80 to 250 °C. The heating and cooling rates were 10 °C min^{-1} and 5 °C min^{-1}, respectively, under nitrogen atmosphere.

1.3 Synthesis of CDI-activated 1,12-dodecanedioic acid (M1)

The procedure was adapted from the literature.[3] 1,12-Dodecanedioic acid (0.0225 mol, 1.0 eq.) was added to a 250 mL round-bottomed flask and suspended in dimethylacetamide (45 mL). CDI (0.0472 mol, 2.10 eq.) was added slowly to prevent excessive heat formation, upon which the solution turned an opaque white, and CO_2 evolution was observed. The mixture was

stirred for an additional 2 hours at 1000 rpm before being filtered *via* vacuum filtration, giving a white powder. The powder was washed with deionised water (10 mL) to remove the by-products (*i.e.*, imidazole), before being transferred to a sealed round-bottomed flask and dried under a vacuum pump at 50°C to remove any trace imidazole/solvent, yielding **M1** (5.6309 g, with an isolated yield of 90.3 %). The purity was calculated by ^1H NMR analysis ($CDCl_3$, 300 MHz) to be 93.8 %, by comparing the ratio of the integrals of the monomer and the DMAc / unreacted diacid peaks. **Note to the reader**: heating the reaction mixture in a warm water bath was required to solubilise the product in $CDCl_3$. ^1H NMR: 8.15 (s, 2H) 7.46 (s, 2H) 7.08 (s, 2H) 2.84 (t, 4H, J = 7.4 Hz) 1.78 (quint, 4 H, J = 7.4 Hz) 1.34 (m, 12H).

1.4 General polythioester synthesis procedure

Into a 5 mL crimp-topped vial, dithiol (1.09 mmol, 1.0 eq.) was weighed along with **M1** (1.09 mmol, 1.0 eq). The vial was sealed and flushed with inert gas (i.e., N_2) for 10 minutes. Subsequently, DBU (2.32 mmol, 2.13 eq.) dissolved in DMAc (0.83 M) from a standard solution was injected while remaining under N_2 flow (1.5 L / min). The mixture was continuously stirred at a rate of 300 rpm. Using a hot plate, the temperature was increased to 65 °C until the mixture of the reactants formed a colourless solution, at which point the temperature was reduced to 50 °C. After 2 hours of stirring, the reaction mixture became highly viscous, inhibiting the stirring of the magnetic stirrer bar, so the temperature was increased to 60 °C to return the appearance of the mixture to a colourless solution. After 1 hour at this temperature, and a total reaction time of 3 hours, the mixture was added dropwise to ice-cold MeOH (30 mL) while stirring vigorously, resulting in a white suspension. The respective mixture was left in a freezer at 0 °C for 16 hours, before being filtered by gravity. The solid was then dried in a vacuum desiccator for 3 hours, yielding a white polymer product (0.2985 g, with an isolated yield of 72.9%). ^1H NMR (300 MHz, $CDCl_3$, **P1**): 2.83 (t, 4H, J = 7.3 Hz), 2.52 (t, 4H, J = 7.54 Hz), 1.58 (m, 8H), 1.35 (m, 8H) 1.25 (s, 12H) ; ^1H NMR (300 MHz, $CDCl_3$, **P2**): 3.58 (m, 8H) 3.07 (t, 4H, J = 6.52 Hz) 2.53 (t, 4H, J = 7.54 Hz) 1.63 (quint, 4H, J = 7.6 Hz) 1.26 (s, 12H). The ^{13}C NMR of the respective polymers after precipitation and dialysis are shown in **Fig. S05** and **S06**.

1.5 Representative post-polymerization modification of P2 into PP2 via thionation agent

Into a 25 mL two-necked flask equipped with a condenser and a rubber septum, **P2** (0.359 mmol, 1.0 eq.) and Lawesson's reagent (0.5385 mmol, 1.5 eq.) were added. Next, analytical grade toluene (1.5 mL, 0.24 M) was added, and the mixture was stirred at 300 rpm while heating under reflux conditions. Upon reaction, the solution turned from dark orange to clear yellow. The reaction progression was monitored *via* FTIR to confirm the conversion of the carbonyl group.

The polymer named **PP2 ~60 %** thionation was obtained after 3 hours by quenching the reaction through cooling the mixture to ambient temperature. The crude product was purified by twice precipitating it into a beaker containing ice-cold methanol (25 mL). The polymer was isolated by decanting the methanol, then dried under vacuum to afford a sticky yellow polymer in 82.8 % yield.

The polymer named **PP2 ~97.6 %** thionation was obtained after 5 hours otherwise identical conditions. The crucial difference was the physical appearance of the polymer, which was a sticky dark orange polymer (0.1091 g, Yield = 70.1%).

Both polymers were further purified via dialysis: The polymer was dissolved in $CHCl_3$ (100 mg/mL), poured into a dialysis tubing (1000 Da, d = 34 mm) and stored in a beaker filled with 400 mL MeOH mL. The surrounding solvent was exchanged every 12 h (3 times) and a new 400 mL of MeOH was utilized. The percentage conversion was calculated *via* ^{1}H NMR (300 MHz, $CDCl_3$) by dividing the integrals of the peaks corresponding to the polythioester and polydithioester. The NMR of the respective polymers after precipitation and dialysis are shown in **Fig. 4C-D** and **Fig. S10.**

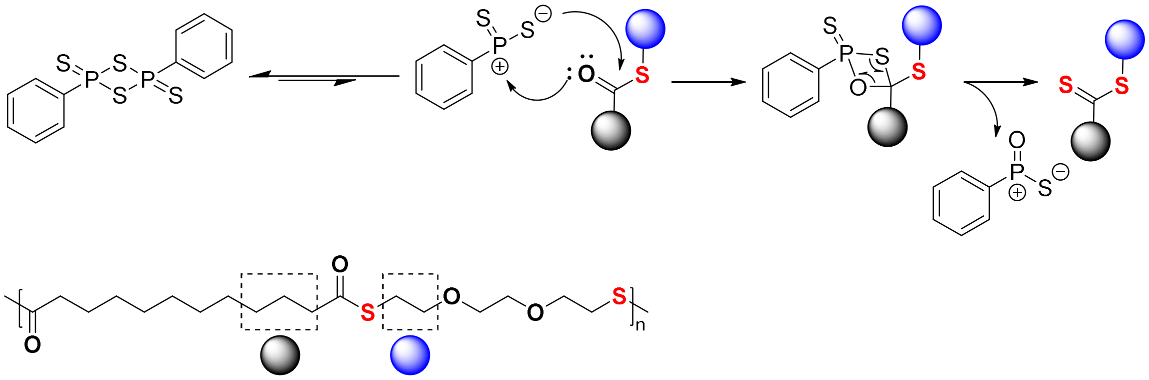

Scheme S01. Reaction scheme demonstrating the post-polymerization modification of polythioester **P2** to polydithioester **PP2**, as reported for the first time in this publication.

2. Figures

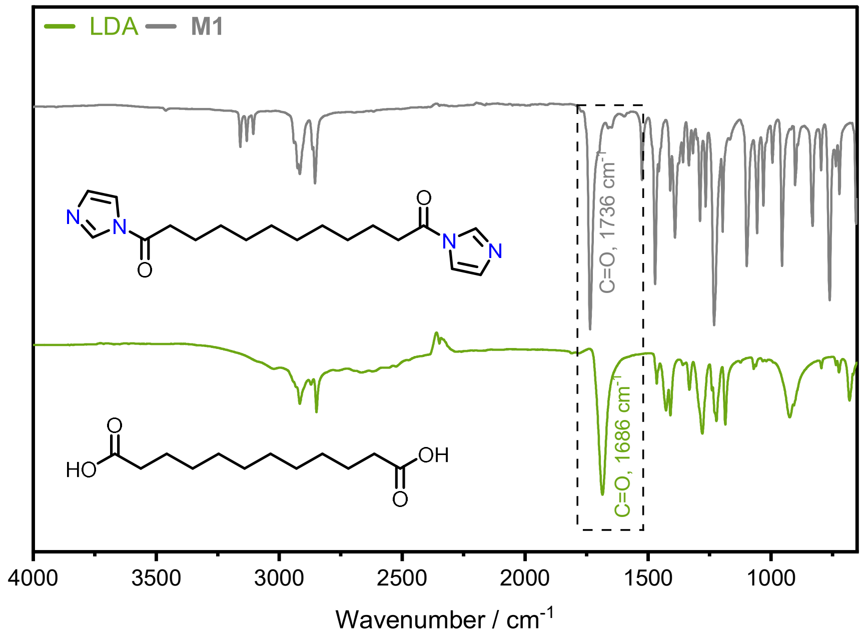

Figure S01. FTIR spectra of the LDA and the CDI-activated monomer **M1**, showing the shifting of the carbonyl vibrational band to a higher wavenumber (from 1686 cm^{-1} to 1736 cm^{-1}) upon activation with CDI.

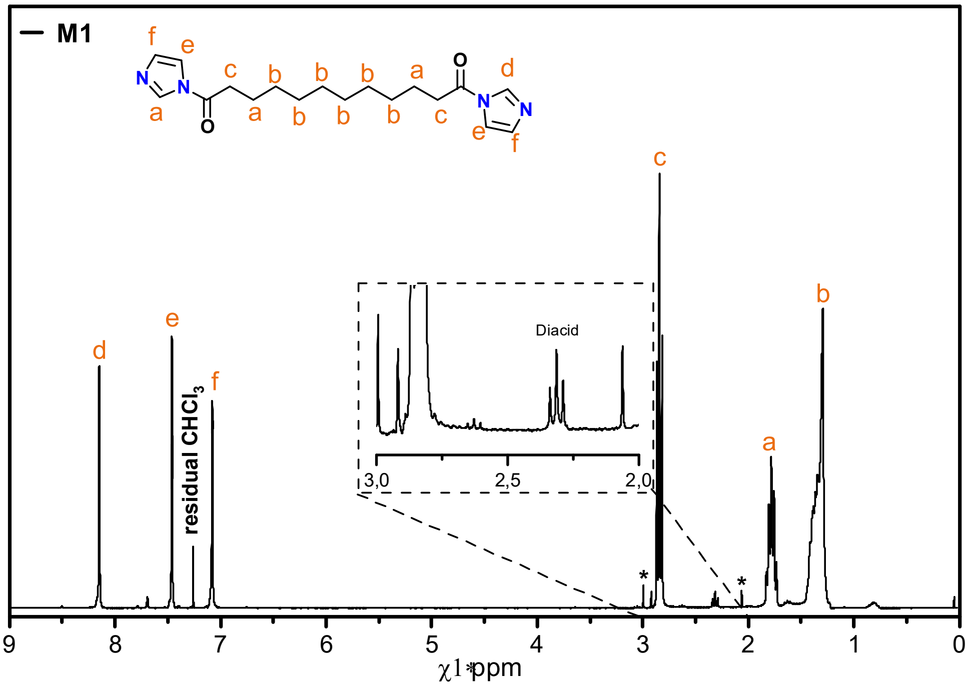

Figure S02. ^{1}H NMR (300 MHz, $CDCl_3$) spectrum of monomer **M1** with proton assignments (a–f). The appearance of additional magnetic resonances in the $\delta = 2.0–3.0$ ppm region indicates partial cleavage of the imidazole moieties, likely forming the corresponding diacid derivative. This degradation occurs under standard NMR conditions, emphasizing the sensitivity of the imidazole group to trace moisture and the need for careful sample handling. Asterisks (*) mark the residual DMAC.

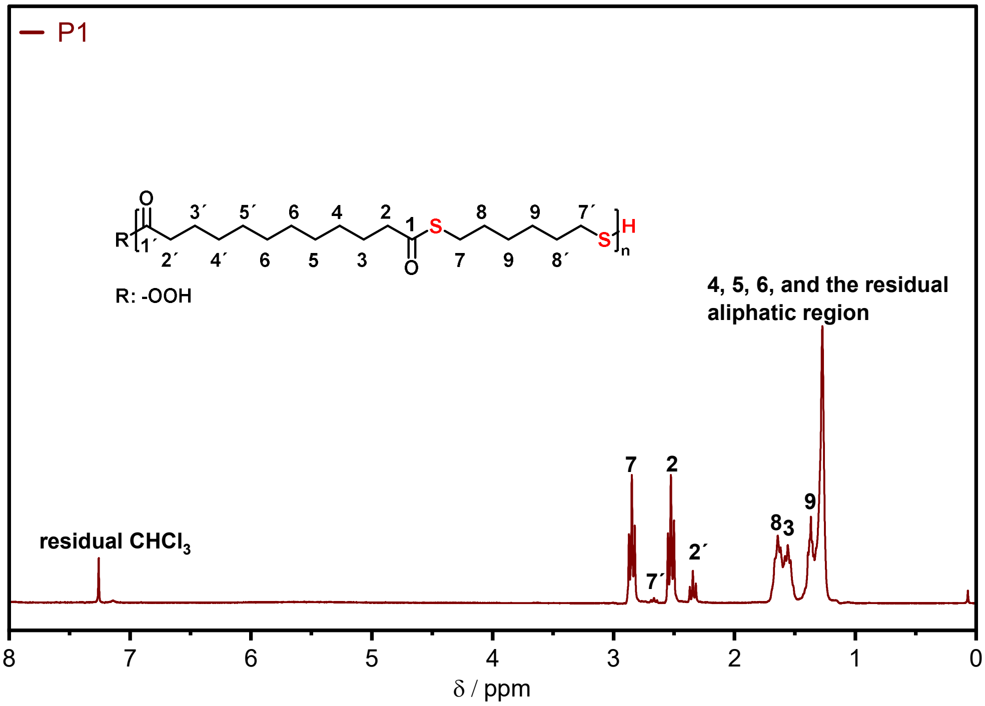

Figure S03. ^{1}H NMR spectrum (300 MHz, $CDCl_3$) of **P1**.

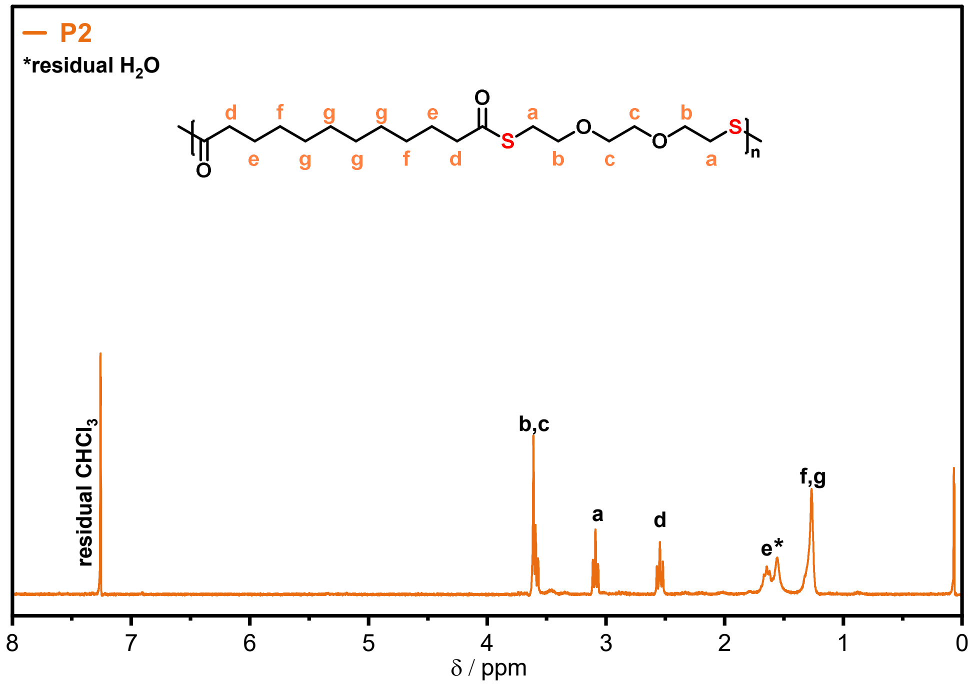

Figure S04. ^{1}H NMR spectrum (300 MHz, $CDCl_3$) of **P2**.

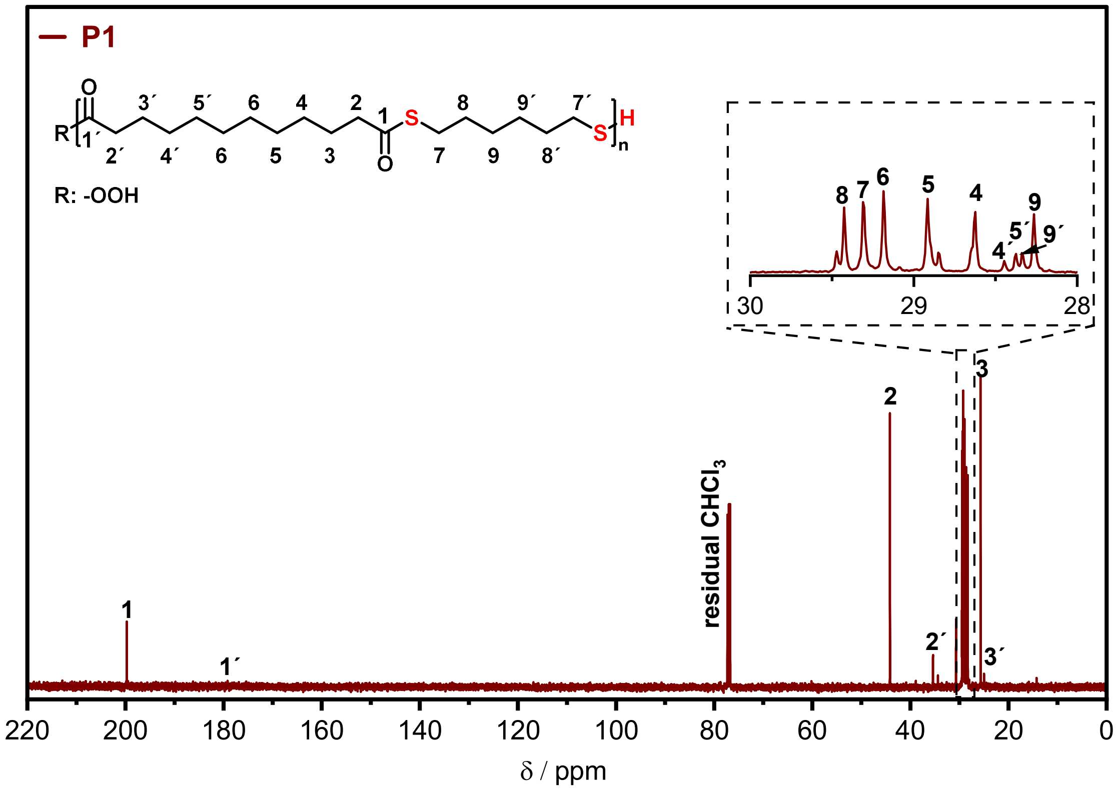

Figure S05. ^{13}C NMR spectrum (75 MHz, $CDCl_3$) of **P1**.

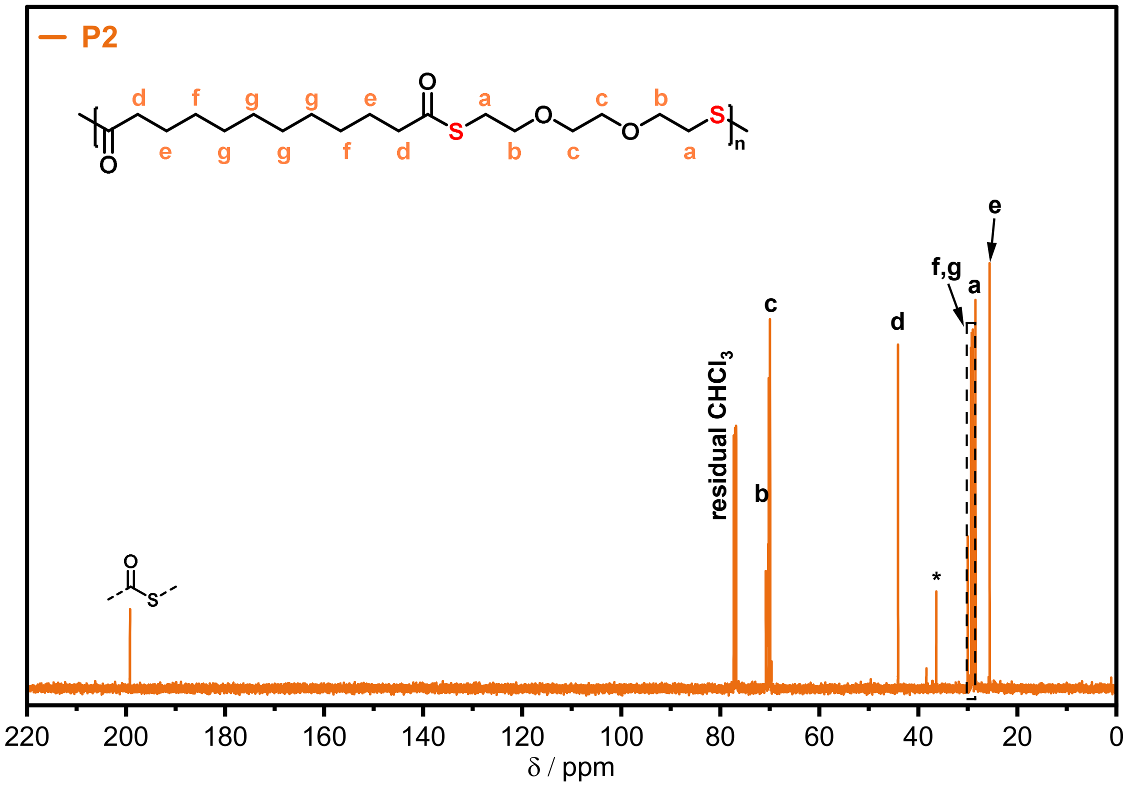

Figure S06. ^{13}C NMR spectrum (75 MHz, $CDCl_3$) of **P2**. The asterisk indicates the residual acetone.

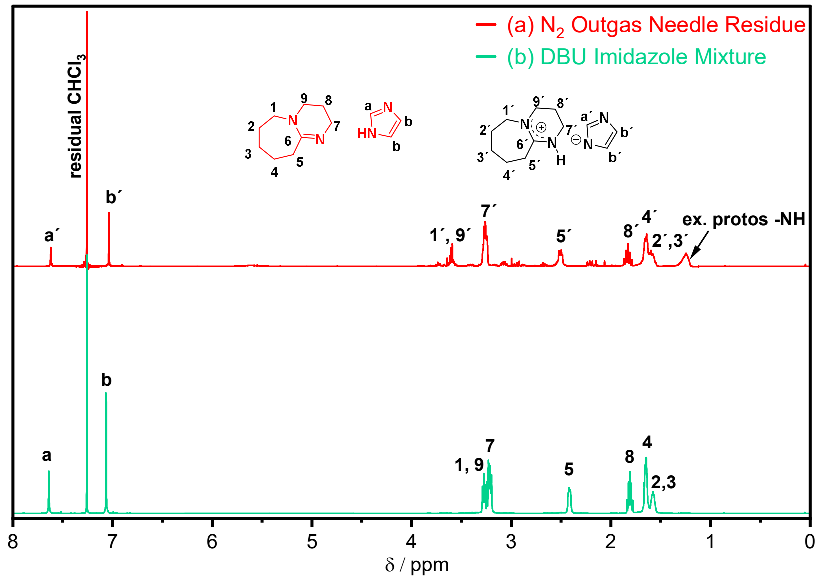

Figure S07. ^{1}H NMR spectra (300 MHz, $CDCl_3$) of (a) the residue deposited on the N_2 outgas needle during the synthesis of polythioesters, and (b) a reference sample of the DBU imidazolium mixture. Note: The sample shown in (a) represents a crude, non-isolated material that accumulated unintentionally on the apparatus surface. It was not subjected to purification, as it lies outside the targeted reaction scope. The observed resonances are consistent with literature reports and suggest the presence of components derived from the DBU–imidazolium ionic liquid.[4]

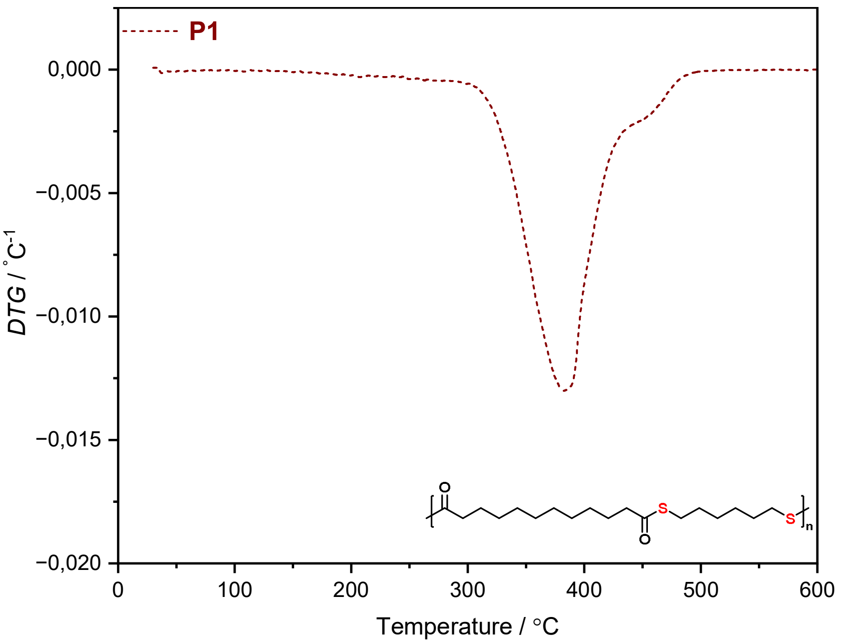

Figure S08 Derivative thermogravimetric (DTG) curve of polymer **P1** showing thermal degradation behavior. A single major degradation step is observed between 380 °C and 450 °C, corresponding to the thermal cleavage of thioester bonds (–C(=O)–S–) within the polymer backbone. The absence of multiple degradation events highlights the thermal integrity and uniform decomposition behavior of the thioester-based structure.

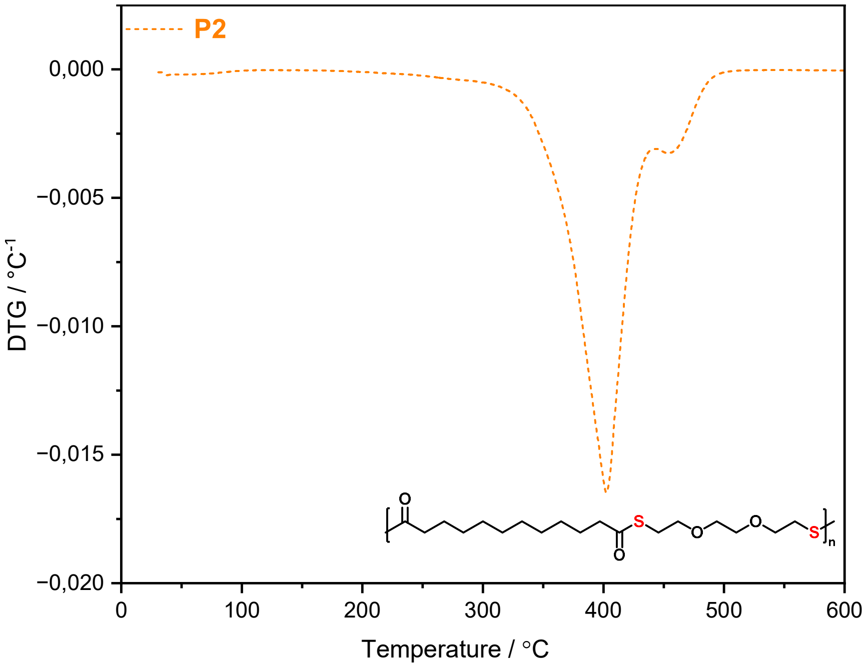

Figure S09. Derivative thermogravimetric (DTG) curve of polymer **P2** depicting its thermal degradation profile. The first major degradation event (~370–430 °C) being attributed to the cleavage of the thermally sensitive thioester bonds within the polymer backbone, followed by a second, smaller degradation step (~480–520 °C) corresponding to the breakdown of the ether segment.

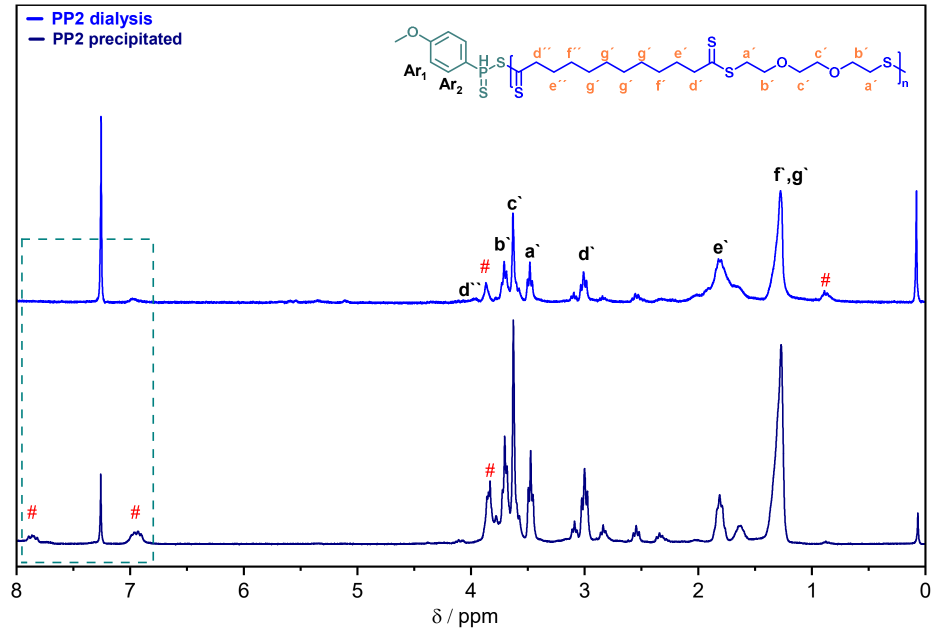

Figure S10. Comparative ¹H NMR spectra (300 MHz, $CDCl_3$) of **PP2** with 97.6% thionation after purification by precipitation in ice-cold MeOH (navy blue) and subsequent dialysis (blue), respectively. The spectra illustrate the effect of purification methods on the chemical profile of the polymer, highlighting the removal of small-molecule impurities and side products.

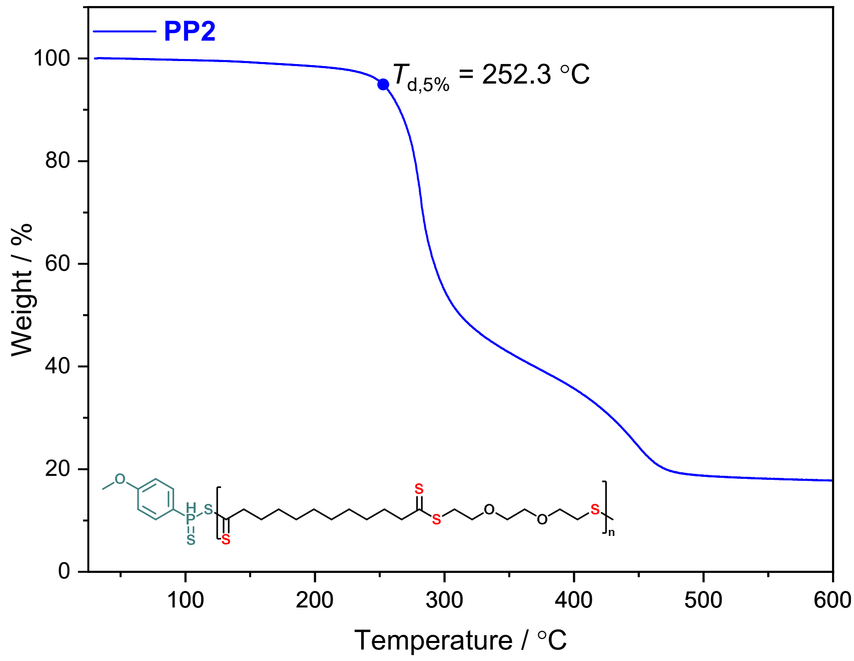

Figure S11. Thermogravimetric analysis (TGA) graph for polythioester **PP2** under nitrogen atmosphere at heating rate of 10 C min^{-1}. Residual mass (~18%) at 600 °C suggests partial char formation.

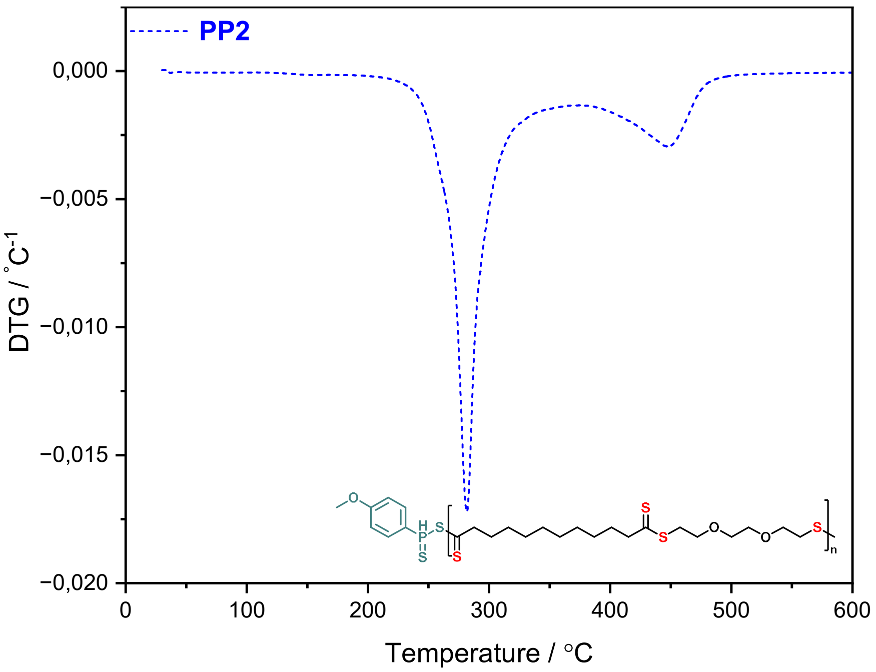

Figure S12. Derivative thermogravimetric (DTG) analysis of the main-chain dithioester polymer **PP2**. The polymer exhibits two distinct degradation stages. The first major degradation step occurs at approximately 300 °C, corresponding to the thermal cleavage of the main-chain dithioester bonds (–C(=S)–S–), which are thermally labile and initiate chain scission. A second, degradation event appearing between 450–500 °C, is attributed to the breakdown of the polyether segments (–CH_2–CH_2–O–) within the backbone. The two-step profile highlights the hybrid thermal behavior of sulfur- and oxygen-containing main-chain functionalities.

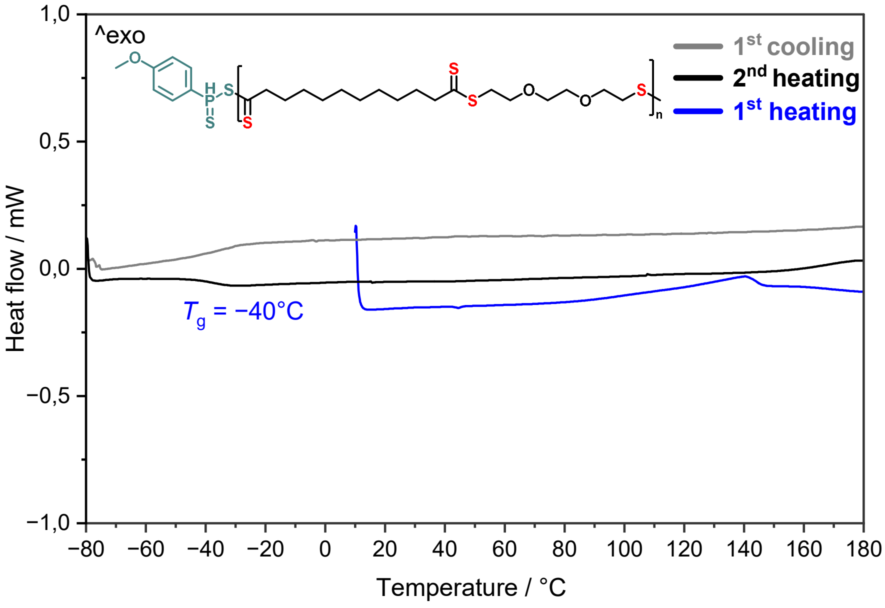

Figure S13. Differential scanning calorimetry (DSC) graph of polythioester **PP2**, showing the 1st heating (blue line), 1st cooling (grey line) and 2nd heating (black line) curves. The heating and cooling scans were performed at rates of 10 °C min^{-1} and 5 °C min^{-1}, respectively.

Purity calculations (based on NMR) for **M1** and conversion calculations for **P1, P2** and **PP2:**

M1: Resonance signals corresponding to DMAc, imidazole and unreacted diacid were present with integrals of 1.68, 2.27, and 5.50, respectively. The monomer had an equivalent resonance with an integral of 100.0. Estimated purity = 100 / (100 + 5.50 + (1.68 * (2/3)) + 2.27 * 2) = 93.8%

P1: Resonance corresponding to polythioester methylene spacer (CH_2) was present at 2.51 ppm with integral of 3.40, while the equivalent diacid methylene spacer (CH_2) resonance had integral of 0.26. Estimated conversion = 3.40 / (0.26 + 3.40) = 92.9%

P2: Resonance corresponding to polythioester methylene spacer (CH_2) was present at 2.53 ppm with integral of 4.13, while the equivalent diacid methylene spacer (CH_2) resonance had integral of 0.28. Estimated conversion = 4.13 / (0.28 + 4.13) x 100 = 93.7%

PP2: Resonance corresponding to polythioester α-methylene showed integral of 0.05, while equivalent polydithioester peak had integral of 2.00. Estimated conversion = 2.00 / (0.05 + 2.00) = 97.6%

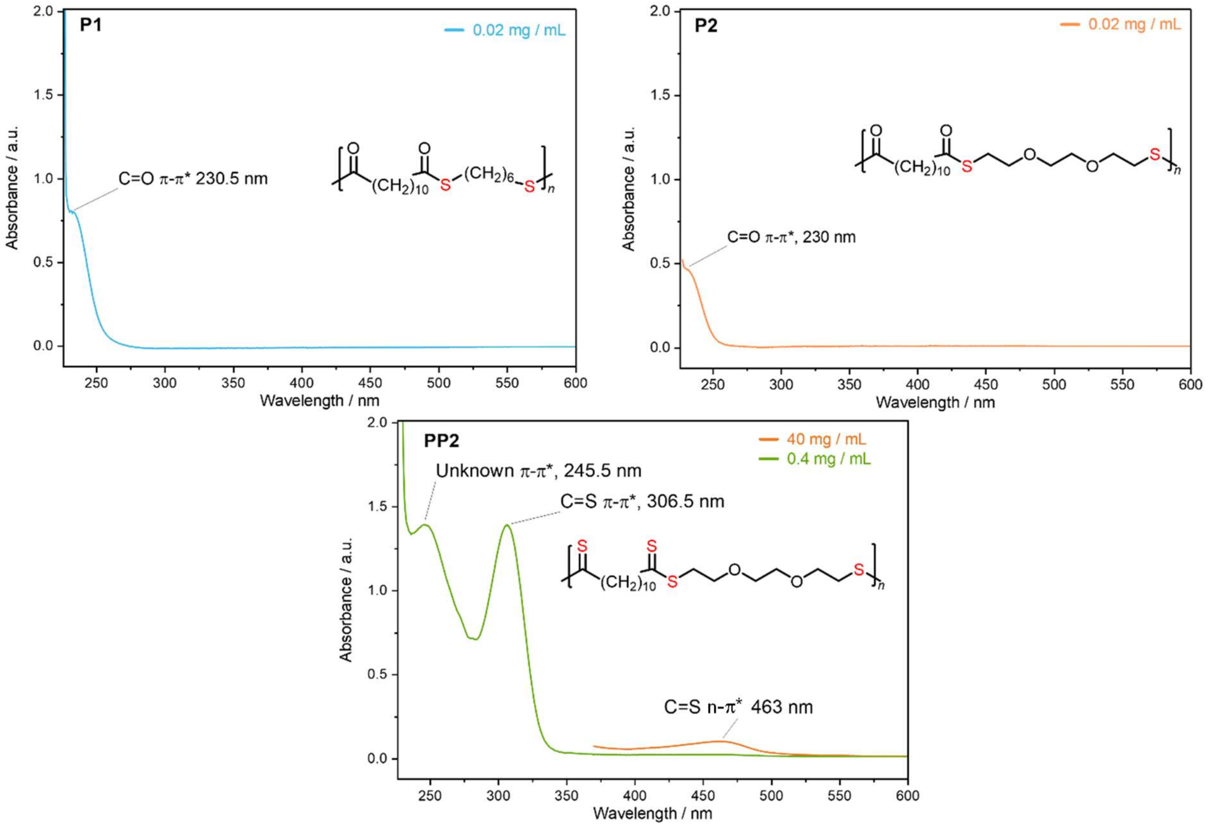

Figure S14. UV/vis spectra (recorded in 40 mg mL^{-1} DCM) of **P1** (top left), **P2** (top right) and **PP2** (bottom), showing the absorption peaks present in each of the spectra.

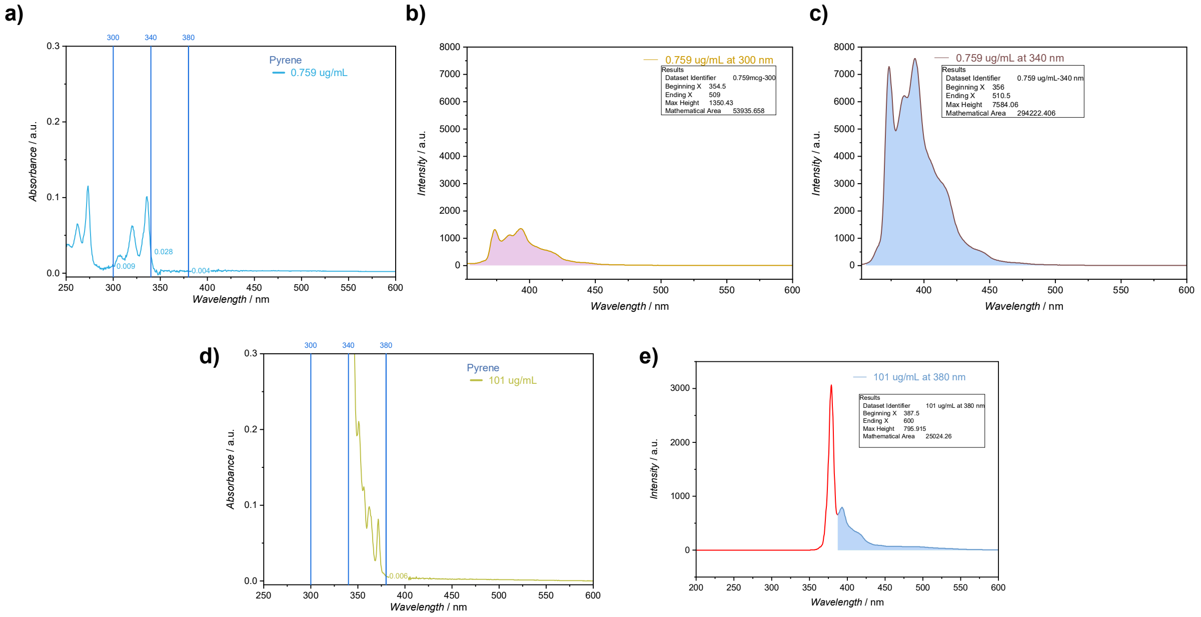

Figure S15. UV-Vis absorption and fluorescence emission spectra of pyrene at two different concentrations, illustrating its characteristic optical behavior and concentration dependence: (a) UV-Vis absorption spectrum of pyrene at 0.759 µg/mL, showing three major absorption bands centered near 300 nm, 340 nm, and 380 nm. These bands correspond to the characteristic π–π* transitions of the aromatic system; (b) Fluorescence emission spectrum of the same 0.759 µg/mL solution, monitored at 300 nm excitation, displaying moderate emission with an integrated area of 53,935 and a peak around 354.5 nm; (c) Fluorescence emission spectrum of pyrene at 0.759 µg/mL, excited at 340 nm, showing a pronounced increase in emission intensity with a max height of 7584 and mathematical area of ~294,222 - highlighting the optimal excitation range for strong fluorescence response; (d) UV-Vis absorption spectrum of pyrene at the higher concentration of 101 µg/mL, where absorption becomes nearly saturated, especially at 340 nm and 380 nm, indicating strong attenuation at higher analyte load; and (e) Fluorescence emission spectrum of pyrene at 101 µg/mL, excited at 380 nm, revealing a highly intense emission signal with a mathematical area exceeding 2.6 million, reflecting strong fluorescence efficiency at elevated concentration.

References

(1) Shirdel, J.; Penzkofer, A.; Procházka, R.; Shen, Z.; Strauss, J.; Daub, J.. *Chem. Phys.* **2007**, *331*, 427-437.

(2) Liang, H.; Zhang, X.; Lu, M.; Chen, X.; Li, W.; Li, S.; Li, M. D.; Zhao, J.; Huo, Y.; Ji, S. *Angew. Chem. Int. Ed. Engl.* **2024**, *63*, e202402774.

(3) M .Glanzmann, C. Karalai, B. Ostersehlt, U. Schön, C. Frese, E. Winterfeldt, *Tetrahedron* **1982**, *38*, 2805–2810.

(4) M. Sharma, K. Verma, A. Kaushik, J. Singh, A. Singh, R. Badru, *Mol. Catal.* **2023**, *536*, 112906.
